# Supplementary material for: Architecture of the Heme-translocating CcmABCD/E complex required for Cytochrome c maturation
Source: Nat Commun. 2023 Aug 25;14:5190. doi: 10.1038/s41467-023-40881-y (PMC10457321; doi:10.1038/s41467-023-40881-y)

Architecture of the Heme-translocating CcmABCD/E Complex required for Cytochrome *c* Maturation

Lorena Ilcu, Lukas Denkhaus, Anton Brausemann, Lin Zhang* & Oliver Einsle*

Institut für Biochemie, Albert-Ludwigs-Universität Freiburg, 79104 Freiburg im Breisgau, Germany

* Correspondence to

lin.zhang@biochemie.uni-freiburg.de

einsle@biochemie.uni-freiburg.de

SUPPLEMENTARY INFORMATION

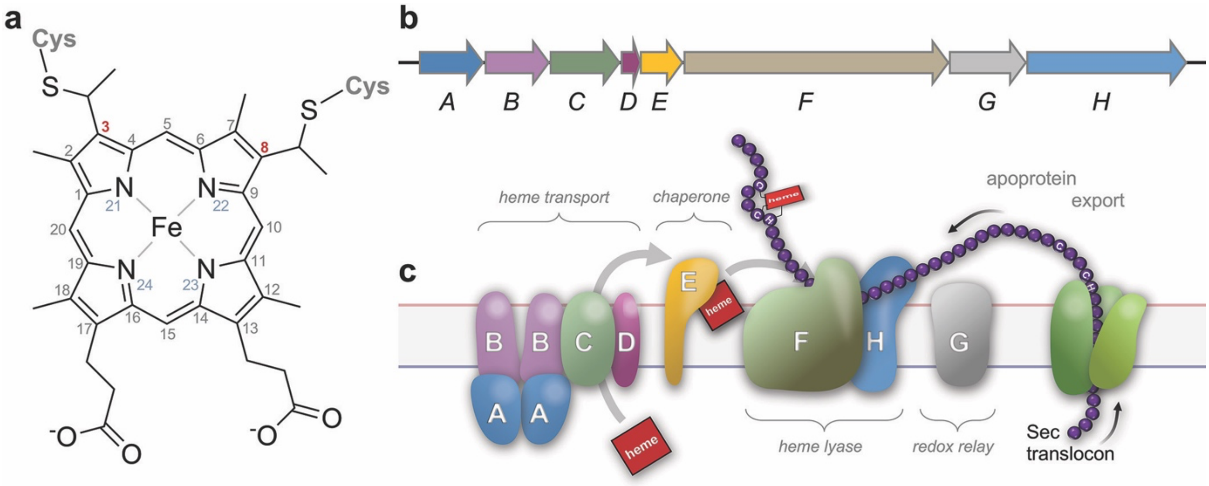

Supplementary Figure 1 | Schematic overview of the system I heme maturation machinery of *E. coli*. **a**, IUPAC nomenclature for Fe-protoporphyrin IX, the heme group, with the covalent links to two cysteine residues from the 3- and 8-position that characterize cytochromes *c*. **b**, Organization of the *ccm* operon. The gene products CcmA, CcmB, CcmC and CcmD form the heme translocase module described here. CcmE is the membrane-associated heme chaperone that receives the cofactor from the heme translocase, and CcmF, CcmG and CcmH form the heme lyase module that scans apocytochromes for CX_nCH motifs, reduces a cysteine disulfide formed in this motif and attaches a cofactor. In many organisms, CcmH is split into two protein products, then designated CcmH and CcmI. **c**, The components of system I in the cytoplasmic membrane (cytoplasm below, periplasm above). Heme groups are flipped from the inner to the outer leaflet by the heme translocase module, and then shuttled to the heme lyase CcmFH by the chaperone CcmE. The apocytochrome chain is exported via the Sec translocon and then scanned by CcmH(I) for binding motifs. CcmG is the component that reduces disulfides in the binding motif in the oxidizing environment of the periplasm.

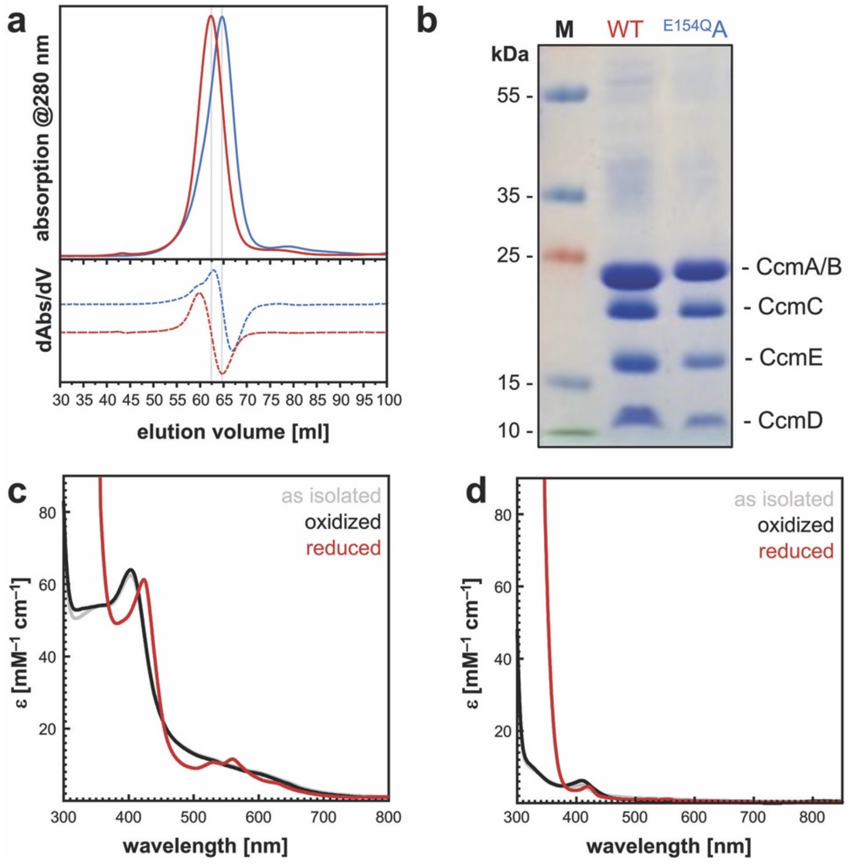

Supplementary Figure 2 | Production, isolation, and characterization of the heme translocase Ccm(ABCD)$_2$(E). **a**, Size exclusion chromatograms for CcmABCD/E wild type (red) and the Ccm^{E154Q}ABCD/E variant (blue) on Superdex 200 (HiLoad 16/600, Cytiva). The difference in peak retention volumes, emphasized in the derivative curves below, is indicative of the loss of one CcmCD module in the variant. **b**, SDS-PAGE of the preparations in (b). Notably, both preparations contain CcmE, although this was a minor species in the 3D reconstructions or entirely absent in the variant complex. All purifications were performed at least three times with highly reproducible outcomes. The panels show a representative SEC profile and SDS-PAGE. **c**, UV-vis spectra of the CcmABCD/E sample from panel (a) as isolated (grey), oxidized with $K_3Fe(CN)_6$ (black) and reduced with $Na_2S_2O_4$ (red). The α-band at 560 nm indicates a heme *b* in the complex. **d**, UV-vis spectrum of the Ccm^{E154Q}ABCD/E complex from panel (b) as isolated (grey), oxidized with $K_3Fe(CN)_6$ (black) and reduced with $Na_2S_2O_4$ (red). Only traces of bound heme are observed. Source Data for **b** are provided.

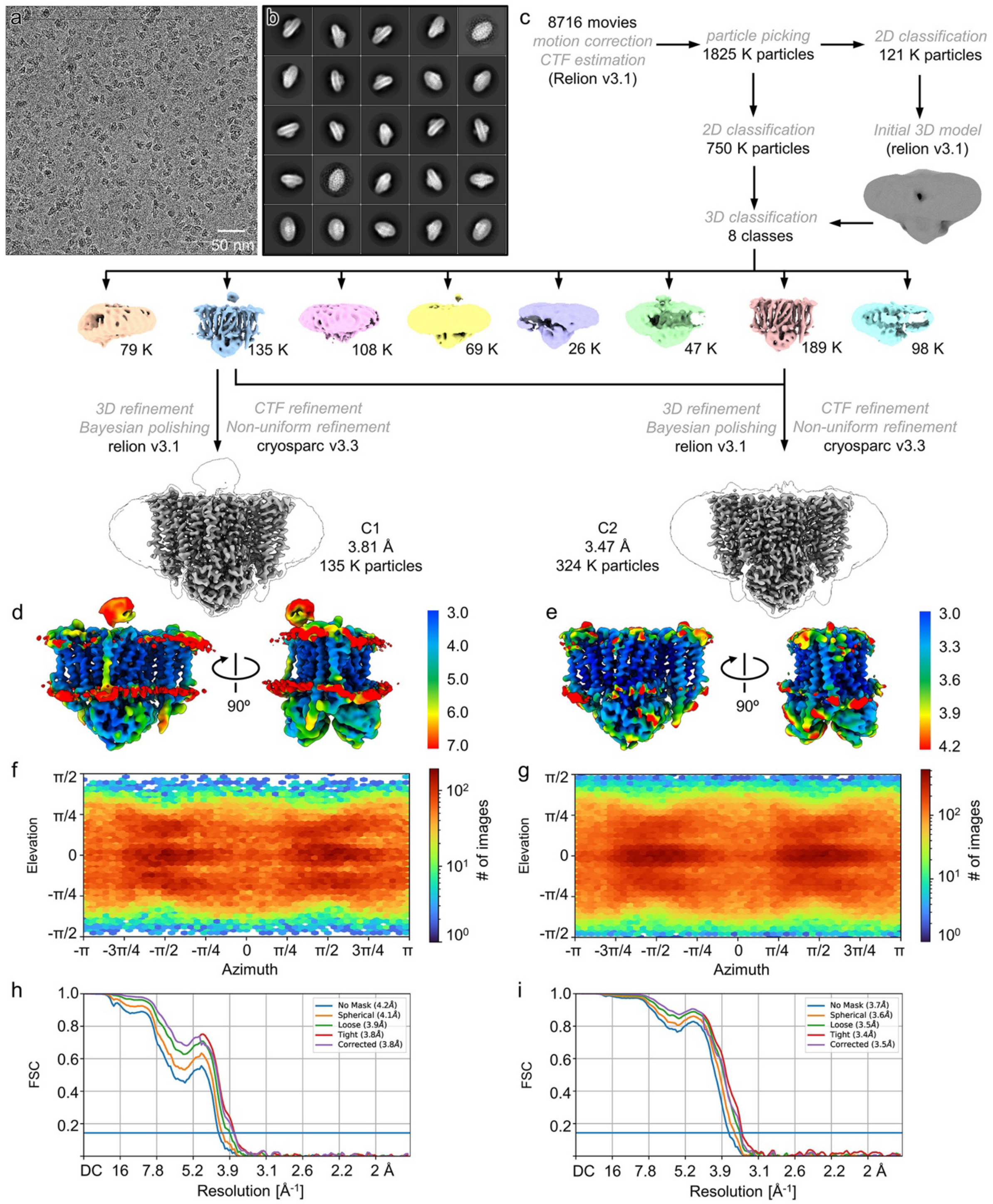

Supplementary Figure 3 | Data Processing Workflow for the Ccm(ABCD)$_2$(E) complex. **a**, representative micrograph, and **b**, 2D class averages from 8716 recorded movies. **c**, refinement workflow and particle numbers for 2D and 3D classification. The two most promising 3D classes were used as starting points for the Ccm(ABCD)$_2$ and Ccm(ABCD)$_2$E complexes. **d**, Local resolution map for the Ccm(ABCD)$_2$E complex. **e**, Local resolution map for Ccm(ABCD)$_2$. **f**, Angular distribution of particles for (d). **g**, Angular distribution of particles for (e). **h**, Fourier shell correlation (FSC) curves for Ccm(ABCD)$_2$E, using the threshold 0.143 to determine the overall map resolution. **i**, FSC curves for Ccm(ABCD)$_2$.

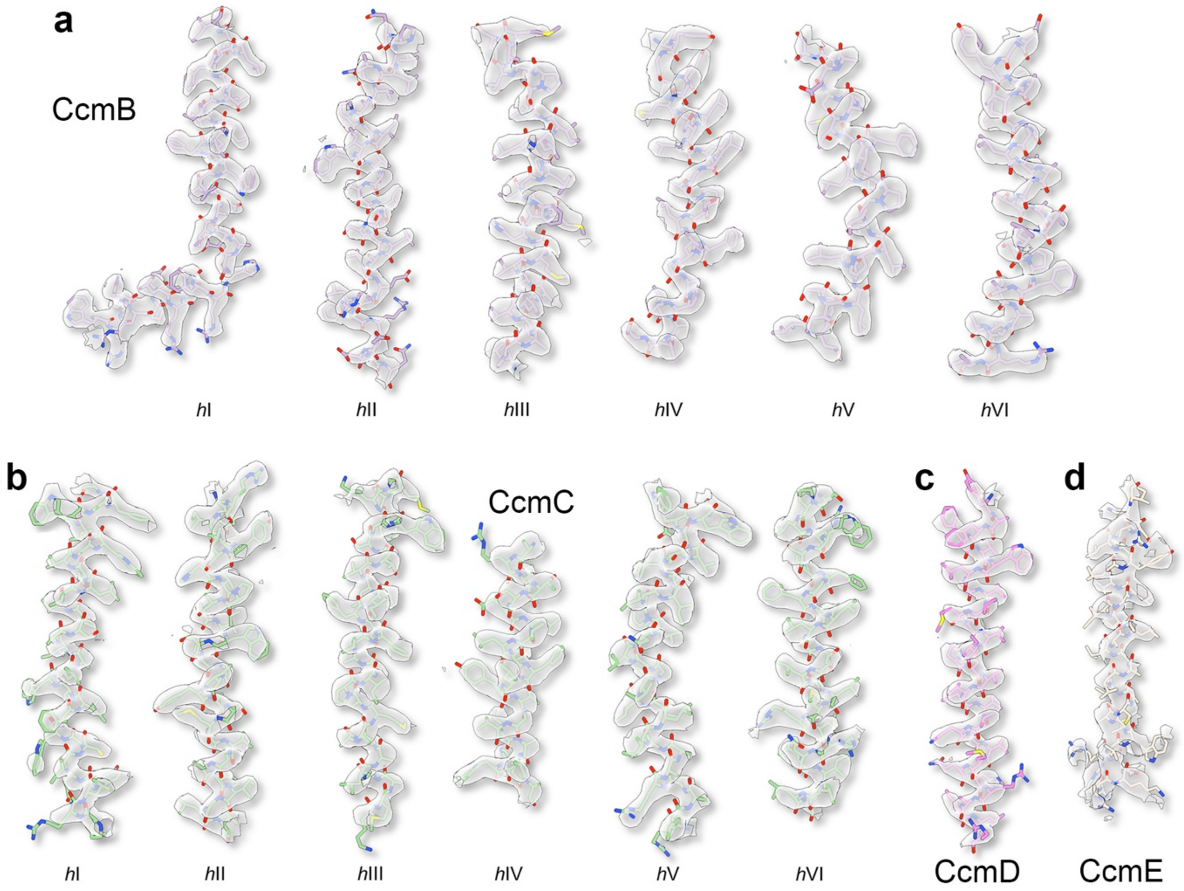

Supplementary Figure 4 | Cryo-EM map quality. **a**, **b**, **c**, Coulomb density maps and model rendered for the transmembrane helices of all membrane subunits of the Ccm(ABCD)$_2$ complex. **d**, Cryo-EM density and model for the N-terminal transmembrane helix of the heme chaperone CcmE in the Ccm(ABCD)$_2$E complex.

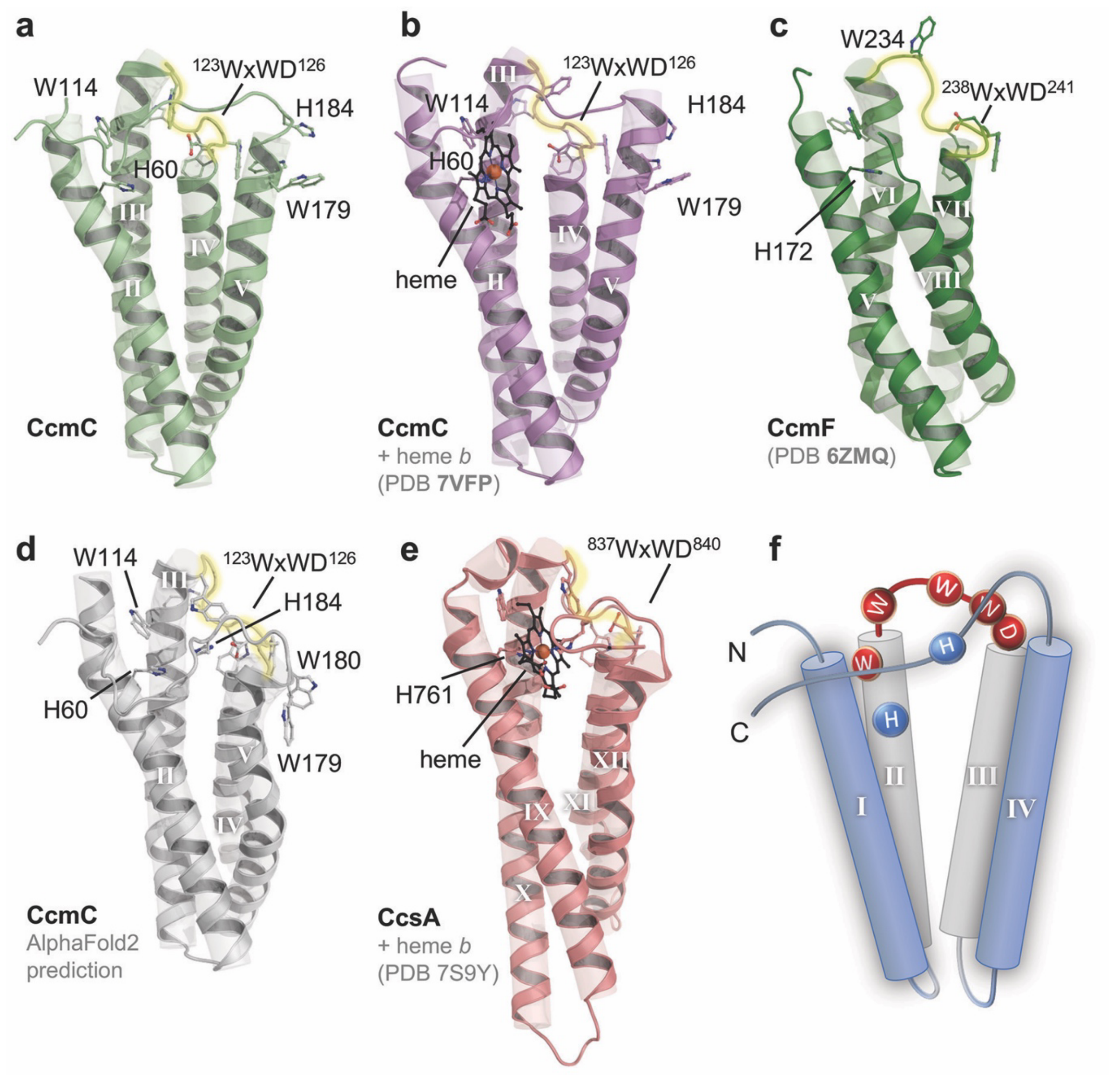

Supplementary Figure 5 | The conserved four-helix bundle around the WxWD motif in heme-handling proteins. **a**, Helices *h*II–*h*V of CcmC in the Ccm^{E154Q}ABCD variant with bound ATP. **b**, Helices *h*II–*h*V of CcmC in the heme-bound CcmABCD structure (PDB 7VFP). **c**, Helices *h*V–*h*VIII of the heme lyase CcmF (PDB 6MZQ), showing a significant rotation of the last helix. **d**, *In-silico* prediction of CcmC by AlphaFold2. Here the loop following helix V and the putative heme ligand H184 are folded back towards the core of the subunit. **e**, Helices *h*IX–*h*XII of *H. hepaticus* CcsA with bound heme (PDB 7S9Y). **f**, Common topology of the four-helix bundle at the core of all known heme-handling proteins. The WxWD motif (yellow) connects the second and third helix on the extracellular side and the histidine ligands to heme are in the second helix and the loop following the fourth.

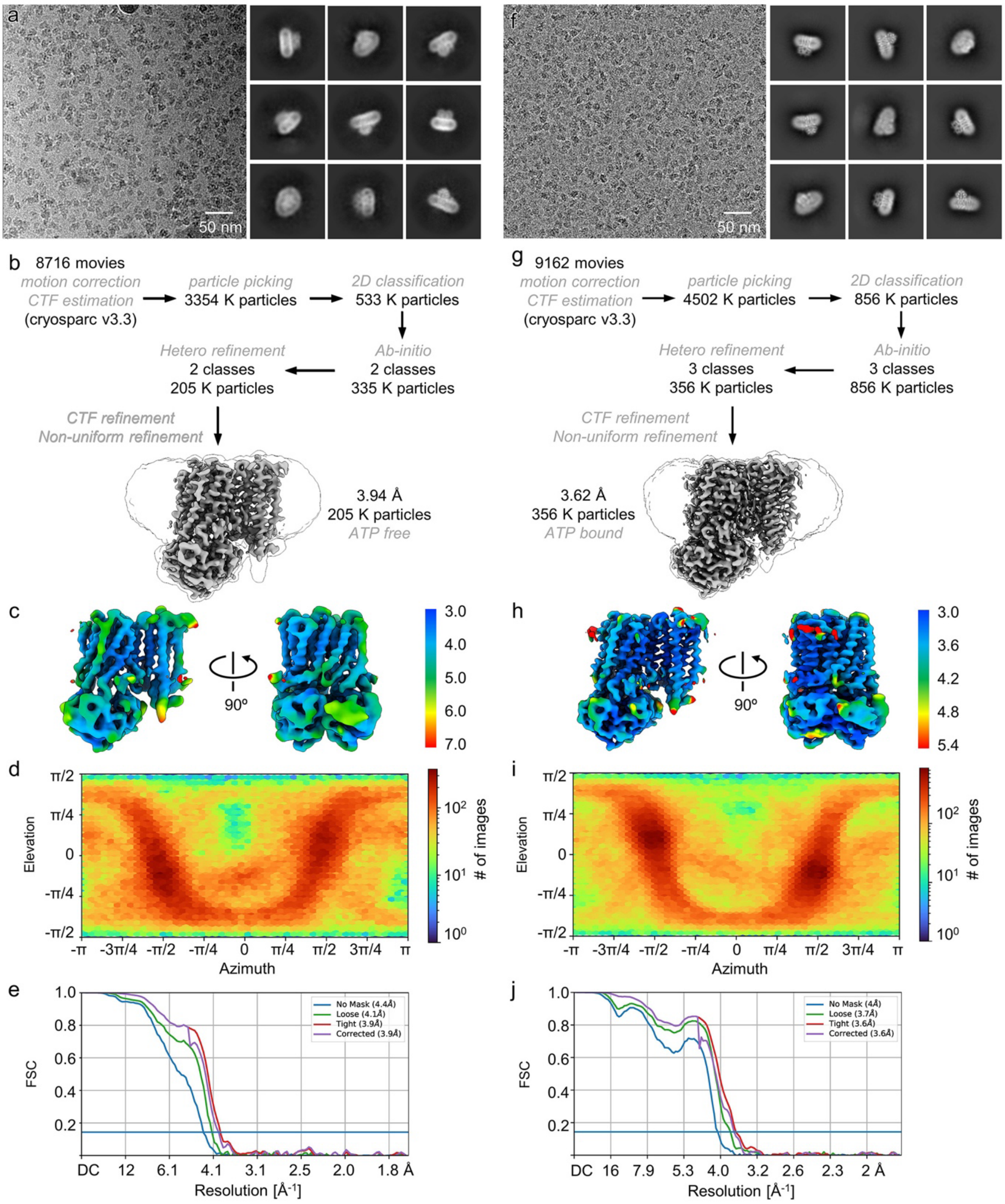

Supplementary Figure 6 | Data Processing Workflow for the $Ccm^{E154Q}(AB)_2CD$ complexes with and without bound ATP. **a**, Representative micrograph and 2D class averages from 8716 recorded movies of the variant complex without added ATP. **b**, Refinement workflow for the ATP-free complex, leading to a refined 3D reconstruction at 3.94 Å resolution. **c**, Local resolution map. **d**, Angular distribution of particles for (c). **e**, FSC curves for (c). **f**, Representative micrograph, and 2D class averages from 9162 movies of the variant complex with ATP. **g**, Refinement workflow, resulting in a 3.62 Å resolution map. **h**, Local resolution map. **i**, Angular particle distribution for (h). **j**, FSC curves for (h).

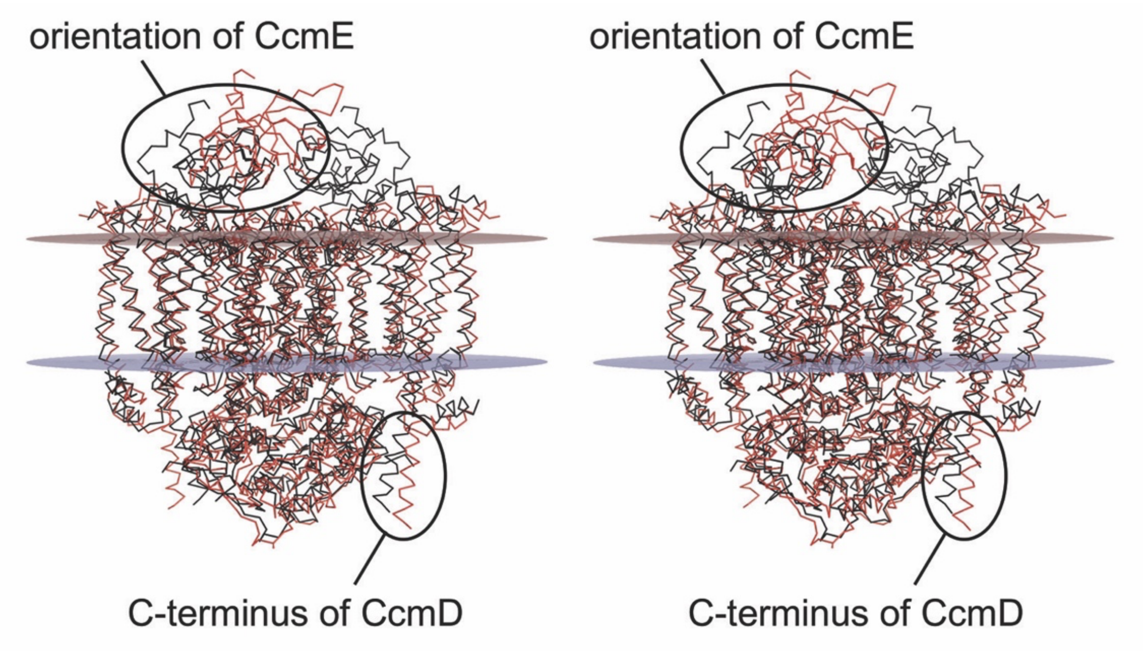

Supplementary Figure 7 | Alignment of the experimental Ccm(ABCD)$_2$E structure with an AlphaFold2 prediction of a symmetric Ccm(ABCDE)$_2$ complex. Stereo image of an overlay of the prediction of a heterodecameric complex made by AlphaFold2 (black) with the experimental structure (red) in ribbon representation (C_α positions only). Both complexes align with an r.m.s.d. of 3.1 Å for all atoms. Major discrepancies were observed for the orientation of the periplasmic domain of CcmE and the C-terminus of the small CcmD subunit.

Supplementary Table 1. Cryo-EM data collection, refinement, and validation statistics.

	WT		E154Q (CcmA)	
stoichiometry	$(ABCD)_2$	$(ABCD)_2E$	$(^{E154Q}AB)_2CD$	$(^{E154Q}AB)_2CD$ + ATP
microscope	Glacios		Titan Krios	Glacios
detector	Gatan K3		Gatan K2	Gatan K3
magnification	46,000		165,000	46,000
voltage (kV)	200		300	200
exposure ($e^-/Å^2$)	1.66		1.66	1.43
defocus range (µm)	-1.0 to -2.0		-1.0 to -2.0	-1.0 to -2.0
pixel size (Å)	0.87		0.82	0.88
number of movies	8,176		7,483	9,162
initial particles	1,825,560		3,988,753	4,502,600
imposed symmetry	C2	C1	C1	C1
final particle number	323,473	135,175	204,806	355,914
resolution (Å)	3.47	3.81	3.94	3.62
FSC threshold	0.143	0.143	0.143	0.143
**Refinement**				
resolution (Å)	3.57	4.06	4.25	3.95
FSC threshold	0.5	0.5	0.5	0.5
r.m.s. deviations in				
bond lengths (Å)	0.006	0.006	0.005	0.008
bond angles (°)	1.082	1.199	1.105	1.288
validation				
MolProbity score	1.50	1.55	1.65	1.72
clash score	6.36	6.92	5.91	7.72
poor rotamers (%)	0.25	0.46	0.00	0.32
Ramachandran plot				
favored (%)	97.16	97.00	95.27	95.68
allowed (%)	2.77	2.93	4.73	4.14
disallowed (%)	0.07	0.06	0.00	0.18

Supplementary Table 2. Bacterial strains, plasmids and primers used in this study.

Strains, plasmids and primers	Relevant characteristics	Ref. or sources
***E. coli* strains**		
XL1-Blue	routine cloning strain, no antibiotic resistance	Stratagene
BL21(DE3)	widely used T7 expression strain	NEB
C43(DE3)	BL21(DE3) derivative containing mutations that prevent cell death associated with expression of many recombinant toxic proteins.	Lucigen
Plasmids		
pEC86	cytochrome *c* maturation helper plasmid, containing the *E. coli* gene *ccmABCDEFGH* with *tet* promoter, resistant to chloramphenicol.	Linda Thöny-Meyer
pASK-IBA5	cytoplasmic *E. coli* expression vector encoding an N-terminal Strep-tag(II), inducible *tet* promoter, resistant to ampicillin	IBA Lifesciences
pASKIBA5-*Ec*Ccm	*E. coli* gene cluster *ccmABCDEFGH* in pASK-IBA5 vector, Strep-tag sequence at the N-terminus of *ccmA*.	this work
pASKIBA5-*Ec*Ccm E154Q	produce mutant E154Q at the ATP-binding site of CcmA.	this work
Primer Names	Sequences	Descriptions
LI-for-pASKIBA5	5' - TGAATTCGGGACCGCGGTCTC - 3'	Gibson, vector
LI-rev-pASKIBA5	5' - CTCGAGGTCGACCTGCAGGG - 3'	
LI-for-Ccm	5' - GAGACCGCGGTCCCGAATTCAATGCTTGAAG-CCAGAGAGTTAC - 3'	Gibson, *ccm*
LI-rev-Ccm	5' – CCCTGCAGGTCGACCTCGAGTTATTTACTCT-CCTGCGGC - 3'	
LI-for-E154Q	5' - GTCGAGGATCCATAACGTG - 3'	E154Q
LI-rev-E154Q	5' – ACGTTATGGATCCTCGACCAGCCTTTTACCG-CGATTG - 3'	

Source Data for Supplementary Figure 2b.

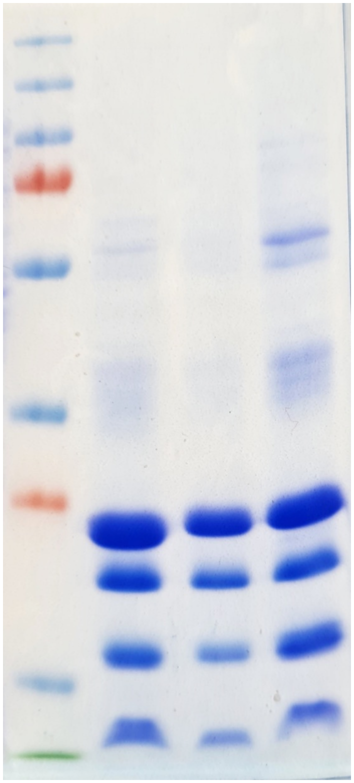

Supplement: Supplementary file 1 — Supplementary Information [file 41467_2023_40881_MOESM1_ESM.pdf]
