# Supplementary material for: Architecture of the Heme-translocating CcmABCD/E complex required for Cytochrome c maturation
Source: Nat Commun. 2023 Aug 25;14:5190. doi: 10.1038/s41467-023-40881-y (PMC10457321; doi:10.1038/s41467-023-40881-y)
Supplement: Supplementary file 3 — Description of Additional Supplementary Files [file 41467_2023_40881_MOESM3_ESM.pdf]

File name: Supplementary Movie 1

Description: Stoichiometry of the CcmABCD heme translocase. The wild type heme translocase complex was solubilized from the membrane as a Ccm(ABCD)$_2$ heterooctamer and characterized by cryo-EM. When a E154Q variant of CcmA was used instead, the core ABC transporter Ccm(^{E154Q}AB)$_2$ attained a closed conformation, but one of the CcmCD units was lost, leading to an asymmetric complex.

File name: Supplementary Movie 2

Description: Conformational changes in the core ABC transporter module and their impact on heme translocation. In the complex, the ABC transporter closes upon ATP binding, but the membrane subunits CcmB do not attain an outward-open form or contain a cavity of sufficient size to accommodate a heme group. Instead, the closing of the CcmB dimer pushes one helix outward. In the structure, this likely triggers the loss of a CcmC unit on one side. Modeling the same change on the other CcmB protomer shows that helix V of CcmB would clash with helix IV of CcmC (Fig. 2g,h), and we suggest that this is a mechanism to couple ATP hydrolysis in CcmA mechanistically to heme translocation along the CcmBC interface.

File name: Supplementary Movie 3

Description: Interaction with the heme chaperone CcmE and comparison with predicted structures. A single copy of CcmE was present in one class of the wild-type data set, showing the interaction of the chaperone with the complex and the positioning of its periplasmic domain. An AlphaFold2 prediction is well in line with this binding mode for CcmE, but has the periplasmic domain rotated by 90°. Together with conformational differences in CcmC, the AlphaFold2 model very well rationalized heme binding to CcmC and a possible handover to the chaperone.
