# Supplementary material for: Architecture of the Heme-translocating CcmABCD/E complex required for Cytochrome c maturation
Source: Nat Commun. 2023 Aug 25;14:5190. doi: 10.1038/s41467-023-40881-y (PMC10457321; doi:10.1038/s41467-023-40881-y)
Supplement: Supplementary file 7 — Reporting Summary [file 41467_2023_40881_MOESM7_ESM.pdf]

nature portfolio

Corresponding author(s): Oliver Einsle, Lin Zhang

Last updated by author(s): Jul 28, 2023

Reporting Summary

Nature Portfolio wishes to improve the reproducibility of the work that we publish. This form provides structure for consistency and transparency in reporting. For further information on Nature Portfolio policies, see our Editorial Policies and the Editorial Policy Checklist.

Statistics

For all statistical analyses, confirm that the following items are present in the figure legend, table legend, main text, or Methods section.

n/a	Confirmed	
☐	☒	The exact sample size (*n*) for each experimental group/condition, given as a discrete number and unit of measurement
☒	☐	A statement on whether measurements were taken from distinct samples or whether the same sample was measured repeatedly
☒	☐	The statistical test(s) used AND whether they are one- or two-sided<br>*Only common tests should be described solely by name; describe more complex techniques in the Methods section.*
☒	☐	A description of all covariates tested
☒	☐	A description of any assumptions or corrections, such as tests of normality and adjustment for multiple comparisons
☒	☐	A full description of the statistical parameters including central tendency (e.g. means) or other basic estimates (e.g. regression coefficient) AND variation (e.g. standard deviation) or associated estimates of uncertainty (e.g. confidence intervals)
☒	☐	For null hypothesis testing, the test statistic (e.g. *F*, *t*, *r*) with confidence intervals, effect sizes, degrees of freedom and *P* value noted<br>*Give P values as exact values whenever suitable.*
☒	☐	For Bayesian analysis, information on the choice of priors and Markov chain Monte Carlo settings
☒	☐	For hierarchical and complex designs, identification of the appropriate level for tests and full reporting of outcomes
☒	☐	Estimates of effect sizes (e.g. Cohen's *d*, Pearson's *r*), indicating how they were calculated

Our web collection on statistics for biologists contains articles on many of the points above.

Software and code

Policy information about availability of computer code

Data collection	SerialEM 3.6
Data analysis	cryoSPARC 3.3; ChimeraX 14.0; Coot 0.9.8; Phenix 1.20; PyMOL 2.5.4

For manuscripts utilizing custom algorithms or software that are central to the research but not yet described in published literature, software must be made available to editors and reviewers. We strongly encourage code deposition in a community repository (e.g. GitHub). See the Nature Portfolio guidelines for submitting code & software for further information.

Data

Policy information about availability of data

All manuscripts must include a data availability statement. This statement should provide the following information, where applicable:

- Accession codes, unique identifiers, or web links for publicly available datasets
- A description of any restrictions on data availability
- For clinical datasets or third party data, please ensure that the statement adheres to our policy

The density maps and atomic coordinates have been deposited with the Protein Data Bank (PDB) at http://www.pdb.org. The cryo-EM density maps were also deposited with the Electron Microscopy Data Bank (EMDB). Accession numbers are: PDB 8CE1 and EMD-16597 (Ccm(ABCD)2), PDB 8CE8 and EMD-16601 (Ccm(ABCD)2E), PDB 8CEA and EMD-16602 (Ccm(E154QAB)2CD), and PDB 8CE5 and EMD-16599 (Ccm(E154QAB)2CD with ATP).

Human research participants

Policy information about studies involving human research participants and Sex and Gender in Research.

Reporting on sex and gender	not applicable
Population characteristics	not applicable
Recruitment	not applicable
Ethics oversight	not applicable

Note that full information on the approval of the study protocol must also be provided in the manuscript.

Field-specific reporting

Please select the one below that is the best fit for your research. If you are not sure, read the appropriate sections before making your selection.

☒ Life sciences ☐ Behavioural & social sciences ☐ Ecological, evolutionary & environmental sciences

For a reference copy of the document with all sections, see nature.com/documents/nr-reporting-summary-flat.pdf

Life sciences study design

All studies must disclose on these points even when the disclosure is negative.

Sample size	3D reconstructions were based on numbers of particles as detailed in Supplementary Table 1. The sample size, i.e. the number of particles included into a given 3D reconstruction, varies during data processing. A large number of particles is automatically picked from all micrographs, and many of these include wrong picks by the algorithm or incomplete or damaged complexes. These are discarded during subsequent stages of the iterative data evaluation process. The flow charts provided in Supplementary Figure 3&4 indicate the number of particles included in each step of the evaluation process.
Data exclusions	As detailed above, the inclusion and removal of individual particle mages is an essential point of the refinement process of a single-particle analysis experiment, as reported in the flowcharts in Supplementary Figure 3&4. Excluded data points are those from the initial auto-picking that were then not used for the final 3D refinement of the cryo-EM maps.
Replication	Production and isolation of the various complexes of CcmABCDE and CcmABCD were fully reproducible, as judged by SEC and PAGE. Cryo-EM dataset collection for a specific sample was generally recorded only a single time.
Randomization	This study does not include any experiments that require randomized samples.
Blinding	This study does not include any experiments that require blinding. However, the datasets in this study were independently processed by different co-authors, partly using different software. In all case, the results were fully consistent.

Reporting for specific materials, systems and methods

We require information from authors about some types of materials, experimental systems and methods used in many studies. Here, indicate whether each material, system or method listed is relevant to your study. If you are not sure if a list item applies to your research, read the appropriate section before selecting a response.

Materials & experimental systems

n/a	Involved in the study	
☒	☐	Antibodies
☒	☐	Eukaryotic cell lines
☒	☐	Palaeontology and archaeology
☒	☐	Animals and other organisms
☒	☐	Clinical data
☒	☐	Dual use research of concern

Methods

n/a	Involved in the study	
☒	☐	ChIP-seq
☒	☐	Flow cytometry
☒	☐	MRI-based neuroimaging
